# Global public health policies: gathering public health associations’ perspectives

**DOI:** 10.1080/16549716.2023.2183596

**Published:** 2023-03-01

**Authors:** Liny Wilson, Sousan Hamwi, Francesca Zanni, Marta Lomazzi

**Affiliations:** a World Federation of Public Health Associations c/o Institute of Global Health, University of Geneva, Geneva, Switzerland; b EPIUnit, Institute of Public Health of the University of Porto, Porto, Portugal; c Department of Biomedical and Neuromotor Sciences, School of Public Health, University of Bologna, Bologna, Italy; d Institute of Global Health, University of Geneva, Geneva, Switzerland

**Keywords:** Public health advocacy, health equity, advocacy, policy, health as human right, COVID-19, SDGs

## Abstract

**Background:**

Advocacy is one of the core functions of public health and is a key tool for achieving Sustainable Development Goals. Public health associations play a key role in advocating for the development and implementation of strategies to prevent diseases and promote health and well-being.

**Objective:**

This study aims to map out the focus of public health advocacy carried out by selected national public health associations over 4 years, between 2018 and 2021, in order to identify gaps and strengths and support associations and professionals in their advocacy efforts.

**Methods:**

Twelve national public health associations participated in the study. Official policy documents produced between 2018 and 2021 were collected and analysed. The title and summary of the policy documents were examined line by line and coded into the main subject categories and themes. A qualitative thematic analysis was conducted. Policies were assessed from global and regional perspectives.

**Results:**

A total of 220 policy documents were analysed. Overall, the largest number of policy documents came from high-income countries and dealt with environmental health and communicable diseases, including COVID-19, with, however, important differences among regions. In the African region, public health advocacy focused mainly on strengthening health systems; Europe and South America were mostly concerned with communicable diseases and pandemic management; and North America and the Western Pacific regions focused primarily on climate change. Limited attention was paid to international health and health as a human right in all regions.

**Conclusion:**

Our study showed that, especially in high-income countries, public health associations actively engage in advocacy; however, more effort needs to be devoted to implementing a more international and intersectoral approach at the global level, anchored in health as a human right and aligned with the Sustainable Development Goals.

## Background

Advocacy definitions vary slightly depending on the discipline [1–5]. The World Health Organization (WHO) defines health advocacy as ‘a combination of social actions designed to gain political commitment, policy support, social acceptance, and systems support for a particular goal or program’ [6]. In 2006, the WHO identified advocacy as a powerful tool for fighting the global epidemic of chronic diseases within public health [7]. Advocacy has also been identified as a core tool for accomplishing the United Nations’ Sustainable Development Goals (SDGs) Agenda 2030 [8].

The SDG’s 17 goals and 169 targets were launched by the UN in 2015 with the aim of eradicating poverty and hunger in all their forms, protecting the planet from degradation, ensuring prosperous and fulfilling lives for all humans, and nurturing peaceful societies worldwide by the year 2030 [9]. Seventeen influential people from around the world have been appointed by the UN Director General as ‘SDGs advocates’, with the mission of raising global awareness about the SDGs and the need for swift action to achieve them [8]. Today, advocacy is considered one of the core functions of public health, and many health organisations devote significant efforts to developing and implementing effective advocacy policies and strategies [10,11].

Advocacy within global public health involves the engagement of diverse stakeholders in the decision-making to improve population health [4]. National public health associations (PHAs) and international non-governmental organisations (NGOs) play a vital role in advocating, advising decision-makers, guiding initiatives, and raising citizens’ awareness [10,12]. Globally, advocacy actions have not been limited to PHAs and NGOs in developed countries; those in developing countries have also shown tremendous achievements in their advocacy efforts [10]. Tools used to influence policy in public health advocacy include, but are not limited to, advocacy initiatives and campaigns, joint position statements, resolutions, and internal policies.

The World Federation of Public Health Associations (WFPHA) is an international NGO comprising the multidisciplinary national PHAs [13]. The Ottawa Charter has highlighted the importance of advocacy for achieving health equity and health promotion [14]. Based on the Ottawa Charter, WFPHA’s Global Charter for the Public’s Health identified advocacy as one of the key determinants in achieving desired public health functions [15,16]. Moreover, a survey study conducted by WFPHA in 2014 has pointed out advocacy as a guiding tool for improving the organisation’s performance [17]. Two of the current goals of WFPHA are advocating for health equity and global policies that improve the health of populations and supporting its members in this endeavour. As advocacy is not a single-step process, the most effective way to attain health equity is when advocacy is adapted to a broad strategy that includes both bottom-up and top-down advocacy [18]. Accurate awareness of WFPHA’s member associations and partners’ foci in public health advocacy is a critical step in achieving global public health goals. The purpose of this study is to map out the focus of advocacy performed by selected national PHAs within the last 4 years to define gaps and strengths that will enable the reinforcement of advocacy activities in favour of health equity and population health, as well as in light of the SDGs.

## Methods

### Study context

The WFPHA is an international professional society representing over 5 million public health professionals worldwide [13]. The Federation has 130 member organisations from around the world, encompassing national and regional public health associations, public health schools, and international partners. Member organisations are present in every continent [13]. With its large number of member organisations, broad regional representation, reputation, and long-standing advocacy work, WFPHA is uniquely positioned to carry out this study.

### Selection of participants

Twenty-two public health associations members of the WFPHA were invited to participate in the study. They have been selected as the most active in advocacy within the corresponding WHO region. To assess each association’s level of activity, the timeliness and frequency of updating their website were used as proxies. Regional federations of public health associations were also consulted when necessary. Public health organisations were contacted between July and October 2021. Email invitations were sent through WFPHA’s points of contact, which are the professionals within each organisation actively involved in WFPHA’s work. Organisations that did not respond were sent up to three reminder emails. Twelve public health associations accepted the invitation and have taken part in the study: those of Ethiopia, Cameroon, South Africa, Nigeria, Canada, the USA, Brazil, Spain, France, the United Kingdom, Australia, and New Zealand.

### Data collection

**Figure 1.**
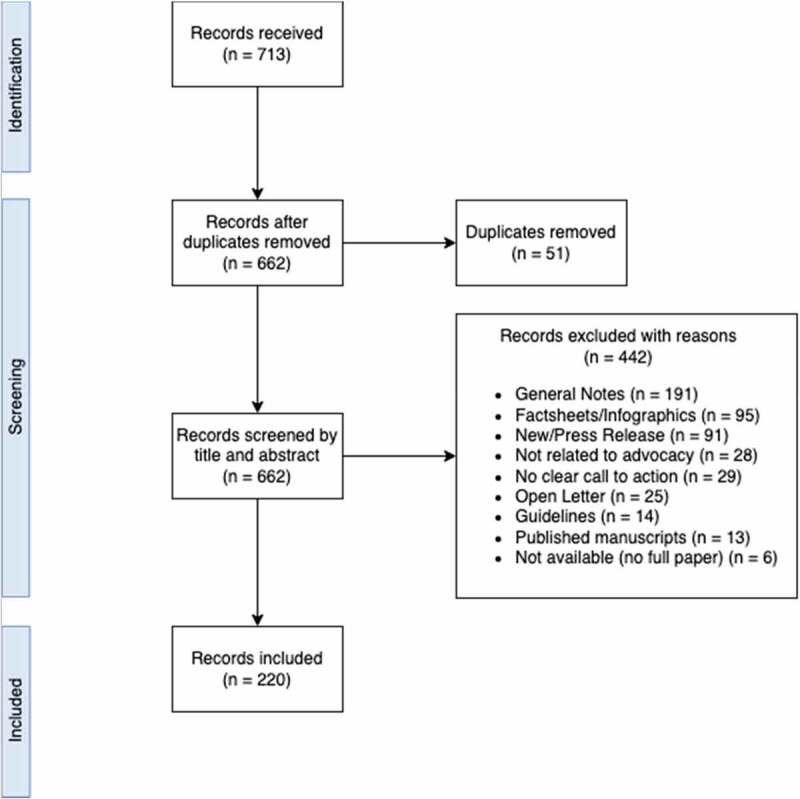
Flowchart of the article selection process.

Upon request, PHAs shared policies were developed between 2018 and 2021. A policy tracking document was created, and policies received were listed by region and country of origin, document name, language, and year of publication. The tracked documents were reviewed by two independent researchers for eligibility. Only documents officially approved by the organisation's board or general assembly were considered eligible for this study. The process leading to official approval might slightly change from one organisation to another, but it usually implies the review of the document by members of the board or the general assembly and the subsequent approval of the final draft through a voting procedure. Documents approved in this way reflect the official stand of the organisation on different matters. Documents considered eligible for our study include: (a) official submissions to government bodies; (b) internal policies; (c) position statements; and (d) white papers. Received documents that were not officially approved by participant organisations include: conference proceedings, guidelines, flyers, infographics, fact sheets, blog posts, news, published manuscripts, press releases, and open letters. These were not considered for this study. The documents submitted in a language other than English (namely, French, Spanish, and Portuguese) were translated using the ‘DeepL’ translator. DeepL is an online translator tool making use of AI technology. It has been rated as one of the most accurate translators in its category. A total of 713 policy documents were screened; 220 met the eligibility criteria and were included in the final analysis (see Figure 1).

### Qualitative analysis

Qualitative thematic analysis was performed by applying the six steps Braun and Clarke’s framework [19]. Such a framework provides clear-cut guidance for ‘identifying, analysing and reporting patterns within data’ [19]. Thematic analysis was chosen as, compared to other qualitative analysis methods, it is rather independent of the theoretical and epistemological traditions [19] and was thus found to be particularly apt to analyse policies. The title and abstract of policy documents were reviewed line by line by two independent researchers, and their topics were coded into main subject categories. As titles and abstracts offer an overview of the topics addressed by policy documents, they provided an appropriate level of information for our study and no further section of the selected documents was analysed. Subsequently, similar categories (Annex A - Table A1) were grouped into themes, and themes were submitted to subject matter experts' input from the WFPHA Policy Committee. They validated the selection of themes as consistent with current trends in advocacy policy discourse.

### Quantitative analysis

Once the qualitative analysis was accomplished, quantitative analysis was undertaken. Proportion calculations were performed to highlight which share of policy documents addressed each theme, both globally and within each WHO region (see Annex A). The combination of quantitative and qualitative analysis allowed the researchers to understand how the themes were distributed across the different regions and to highlight common points and discrepancies.

## Findings

### Quantitative findings

**Figure 2.**
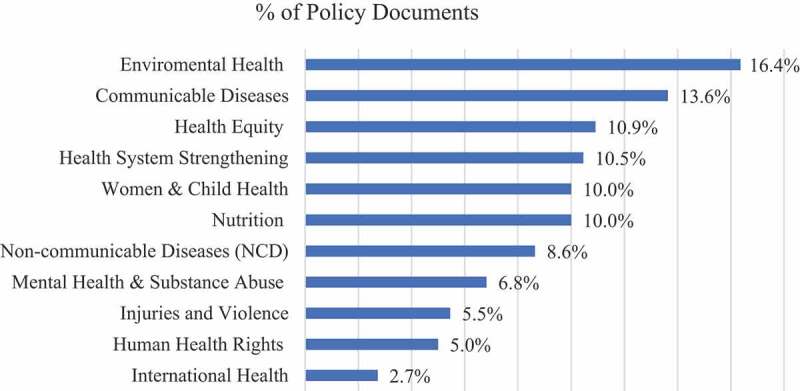
Distribution of global themes across policy documents.

The majority (81.4%) of analysed documents were from high-income countries, namely Australia, Canada, France, New Zealand, Spain, the United Kingdom, and the United States of America, 14.5% of policy tools were retrieved from upper-middle-income countries (Brazil and South Africa), while only 4.1% came from low-middle-income countries (Cameroon, Nigeria) and low-income countries (Ethiopia) [20]. At the global level, 11 key themes concerning public health advocacy emerged (Figure 2): ‘Communicable Diseases’, ‘Environmental Health’, ‘Health Equity’, ‘Human Health Rights’, ‘Health System Strengthening’, ‘Injuries & Violence’, ‘International Health’, ‘Mental Health & Substance Abuse’, ‘Non-communicable Diseases’, ‘Nutrition’, ‘Women & Child Health’.

**Figure 3.**
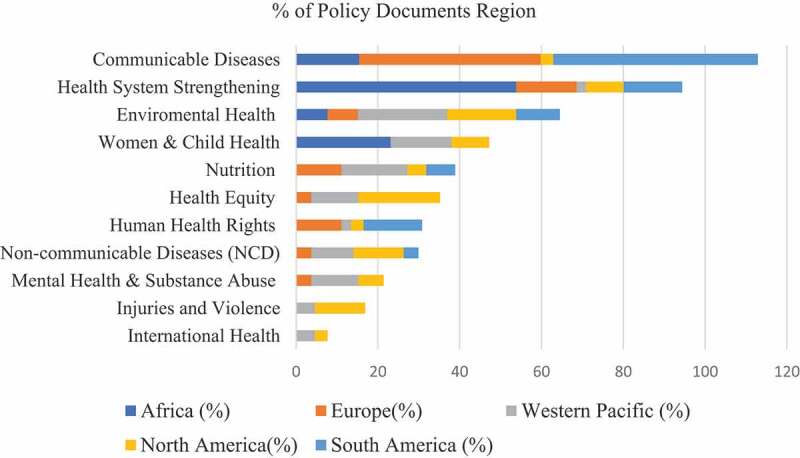
Distribution of themes across policy documents by region.

The global analysis was followed by a regional analysis to understand how the themes were distributed across different regions (Figure 3). In the African region, the primary focus of public health advocacy was on ‘Health System Strengthening’ (53.8% of documents). While Cameroon, Ethiopia, and South Africa focused on this area, Nigeria has carried out most of the advocacy work around ‘Women & Child Health’. Other areas of advocacy focused on ‘Communicable Diseases’, namely COVID-19.

In Europe, most public health advocacy policies focused on COVID-19 (44.4%). Spain had the highest policy production focusing on COVID-19, followed by the UK and France. The second most common advocacy objective was ‘Health System Strengthening’ (14.8%). ‘Nutrition’ and ‘Human Health Rights’ ranked third (11.1% each), with most documents coming from the UK and Spain. Other topics of European interest were ‘Environmental Health’ (7.4%), ‘Health Equity’, ‘Non-Communicable Disease’, and ‘Mental Health & Substance Abuse’ (3.7% each).

In the Western Pacific region, the most common themes were ‘Environmental Health’ (21.8%), ‘Nutrition’ (16.1%), ‘Women & Child Health’ (14.9%), and ‘Mental Health & Substance Abuse’ and ‘Health Equity’ (11.5% each). Policies on ‘Non-communicable Diseases’ were also produced (10.3%), while ‘International Health’ (4.6%) and ‘Injuries & Violence’ (4.6%) were less commonly addressed. A few policies on ‘Human Health Rights’ and ‘Health System Strengthening’ were also identified.

Our results showed significant differences between North (Canada and USA) and South (Brazil) America, thus an in-depth analysis of the two regions separately was conducted. In North America, policies mainly addressed ‘Health Equity’ (20%) and ‘Environmental Health’ (16.9%). The themes of ‘Non-communicable Diseases’ and ‘Injuries & Violence’ ranked third (12.3% each), followed by ‘Women & Child Health’ and ‘Health System Strengthening’ (9.2% each). Less commonly discussed themes included ‘Human Health Rights’, and ‘International Health’. In the South American region (Brazil), half of the policies focused on ‘Communicable Diseases’, including COVID-19 and other infectious diseases like HIV/AIDS. ‘Health System Strengthening’ and ‘Health as Human Right’ ranked second (14.3% each), while few policy documents regarded ‘Environmental Health’, ‘Non-communicable Diseases’, and ‘Nutrition’.

### Qualitative findings

The African region has heavily directed advocacy efforts towards health system strengthening. In Cameroon, policies have advocated for developing effective communication strategies for primary and secondary prevention of COVID-19, while also emphasising the need for revitalising primary healthcare through the establishment of health districts, integration of vertical health programs, and training of human resources. Similarly, the importance of involving economic actors to improve financial investments in the region was stressed. Ethiopia discussed the effect of COVID-19 on healthcare delivery and advocated for effective communication with various stakeholders, implementing think tanks, improving management skills, linking data to policy formulation, and performing high-quality research. South Africa highly advocated for Universal Health Coverage and the reduction of healthcare expenditures.

In Europe, the UK, Spain, and France have widely advocated for measures against COVID-19. Before the pandemic, the call to action to establish a secure and sustainable food system was the main advocacy priority in the UK. Our findings indicate that, due to COVID-19 and the lack of emergency planning, the food system is further weakened in the country. Recommended actions included: a stronger role for local government in the provision of food in both crisis and normal times, population-level health assessment to ensure food provisions, and paying attention to the needs of at-risk groups to reduce dietary inequalities. In Spain, policies on COVID-19 suggested paying special attention to vulnerable groups, particularly elderly people in nursing homes, front-line workers, people living in poverty, and other active workers in various sectors. Policies in Spain also stressed the need for good governance, effective coordination, and health administration for pandemic management. In France, advocacy activities aimed at COVID-19 management in isolated people by offering psychological support and deploying COVID-19 mobile teams.

Advocacy in the Western Pacific mainly focused on environmental health. In Australia, considerable advocacy aimed at decreasing reliance on fossil fuels, highlighted the need for improved funding for research in renewable energy, and recommended abolishing the nuclear power industry in the country and decreasing environmental noise pollution. The concept of ‘One health’ was well addressed by proposing recommendations to support sustainable farming practices and incorporating a ‘planetary health approach’ to public health. Environmental policy documents from New Zealand addressed safe water access, climate change, and transport and health. Nutrition was also addressed by Australian policies, suggesting measures for food safety, obesity, and a healthy diet. Policies regarding women and child health in the region focused on abortion, breast cancer screening, breastfeeding, and improving sexual and reproductive health. Health Equity was a hot topic both in New Zealand and Australia. The former was concerned with budget policies to reduce health inequality and bills to improve living standards, while the latter dealt with gender health and policies to promote health equity in the country.

The main focus of advocacy in North America was Health Equity. Policies in this field (mainly from the US) extensively discussed improving the health of Indigenous populations, migrant and refugee health, prison health, geriatric health, and measures to prevent racism. Advocacy in both the US and Canada also had a strong focus on Environmental Health. In the USA, policies declared the climate crisis a ‘health emergency’ and made ‘environmental justice’ a top priority. The findings were indicative of adopting science-based targets, developing incentives for the use of low-carbon care pathways, enforcing strict laws for coal mining with regular updates on health safety, and setting up stronger science-based ‘National Ambient Air Quality standards’. Shifting to renewable energy sources like solar, wind, hydro, and geothermal were highly recommended as long-term options. Canada has suggested phasing out fossil fuel subsidies and encouraging environmentally sound innovations in the agricultural sector as part of its advocacy for climate change.

Communicable diseases were the main area of policy discussion in Brazil. Policy suggestions to prevent HIV/AIDS included the need for a quality and universal offer of services, educational campaigns, and ensuring human rights. The main priority in advocacy on COVID management was the inclusion of race/colour information in the ‘COVID-19 information system’, in order to reduce racial inequalities. Other policies advocated for expanding hospital beds, funding emergency research on COVID-19, implementing telemedicine, and protecting the rights of Indigenous people.

## Discussion

The purpose of this study was to map the direction of public health advocacy by WFPHA member national PHAs over the past few years, to identify strengths and gaps in national and international advocacy and highlight areas where policy development could be strengthened. We found that the majority of policy documents collected were from high-income countries. Lack of resources and competing priorities could be factors limiting PHA’s advocacy in less wealthy countries. Overall, most policies focused on environmental health and communicable diseases, including COVID-19 and some other infectious diseases. The Western Pacific and North America, more specifically, had an important focus on environmental health, a key issue for countries that rank low in climate protection [21].

Some of the policies included in this review featured a call to action to end wars or conflicts, or regarded international trade agreements. These themes were classified as ‘international health’. Compared to global health, international health is concerned with ‘health of participating countries with intention to affect non-participating countries, while global health deals with health promotion, prevention and treatment of diseases’ [22]. Policy documents addressing international health and health-related human rights were very few, which could highlight a skewed view of international affairs by public health professionals.

At the regional level, we observed significant differences in how advocacy around COVID-19 has been carried out. In some European countries like Spain, the UK, and France, as well as in South America, COVID-19 has been the focus of policy development, while the Western Pacific region had no policy documents on COVID-19. A similar situation has been observed in North America, where PHAs have not been widely advocated, through official policies, for preventive measures to contain COVID-19. This approach may be explained by the fact that advocacy around the pandemic in these regions has been carried out using different communication channels and tools rather than official policies [23–25].

In the African region, the focus on health systems strengthening fits well with the current situation of the African health system, which is characterised by a lack of funding, available human resources lacking the appropriate skills, capacity, and resources to provide quality service, and a weak management system [26]. The WHO African Region has proposed using a comprehensive ‘health system strengthening framework’ to address the peculiar challenges facing the health system in these regions [27,28]. Regarding the SDGs, Africa is facing challenges in addressing the need for good health and well-being (Goal 3), infrastructure (Goal 9), and peace, justice, and institutions (Goal 16) [29]. This study highlights the fact that countries in the African region have discussed the effect of COVID-19 on the health system; it is likely that the considerable impact of COVID-19 on Africa has further slowed down the region’s efforts to achieve the SDGs [29]. Investments in Universal Health Coverage and resilient health systems are more than ever needed to ensure the health and wealth of the region [30].

In the European region, where countries struggled during the initial phase of the pandemic [31], communicable diseases, particularly COVID-19, were the main subject of policy and communication campaigns. In addition, the European region is concerned about environmental health risks and unhealthy behaviours, such as smoking, poor diet, obesity, lack of physical activity and alcohol consumption [31]. Indeed, the region faces major challenges in achieving SDGs 2, 12, and 15 which include sustainable diets, climate, and biodiversity, respectively, [32]. The results of this study indicate that few policy suggestions have been made to prevent addictive behaviours such as gambling, restrictions on the sale of alcohol to minors, food safety, and air pollution, suggesting the need for more advocacy addressing these public health challenges. Nevertheless, in the past, several public health associations based in the European region have highlighted achievements and advocacy activities on their respective websites [17].

In the Western Pacific region, our study reveals a high level of advocacy on environmental health, nutrition, women and children’s health, and health equity, with significant efforts to reduce health inequalities. These results are consistent with the progress made in recent years in health and well-being (Goal 3), infrastructure (Goal 9), sustainable cities (Goal 11), and tackling climate change (Goal 13) [33]. Despite progress in these areas, many Pacific countries still need to focus on the SDGs for responsible consumption and production (Goal 12), peace, justice, and strong institutions (Goal 16) and progress on the partnerships for the goals (Goal 17) [33]. According to the WHO Western Pacific region reports, the greatest concerns for future health regard health security, non-communicable diseases, the health consequences of climate change, children and women’s health, and infectious disease control [34]. The region is currently using innovative approaches by investing in data sources to address these health challenges [33].

In the North American region, the most discussed issues were health equity and environmental health. Specifically, in the US, progress has been very slow in addressing climate change and achieving the SDGs related to ending poverty (Goal 1), health and well-being (Goal 3), sustainable cities (Goal 11), and peace, justice, and institutions (Goal 16) [35]. Data obtained in this study reveal only a few policy documents addressing mental health problems, infectious diseases, nutrition, health as a human right, and international issues, highlighting a gap to be filled by national PHAs and civil society in general.

The South American region focused mainly on policies to control the COVID-19 pandemic. Policies showed how Brazil experienced problems in terms of preventive measures during the pandemic due to a lack of resources and several other obstacles. The shift in policy focus towards COVID-19 has raised concerns about other infectious diseases on the rise in South America, such as malaria, which has very high mortality rates [36]. Public health advocacy in Brazil has also focused on strengthening the health system, mainly discussing policies to reform the primary health care system. To strengthen the health system, accountable data is needed although the country collects a large amount of digital health data, it lags behind the OECD (Organisation for Economic Co-operation and Development) countries in terms of data collection and reporting, and efforts are needed in building efficient health information infrastructure and surveillance mechanisms to identify the health needs of the population [37].

## Limitations

This study involved selected public health associations in four regions. Although the results provide valuable insights into public health advocacy, broader approaches covering other regions and countries are needed to obtain a comprehensive overview. Furthermore, only the most active organisations in each region have been invited to take part in the study. This choice was made in order to gather a picture of the main direction advocacy was taking in the selected regions. However, advocacy efforts of less active organisations, which were not included in the study, might focus on different areas. Thus, our results must not be interpreted as describing all advocacy carried out in the selected regions.

The timeliness and frequency of the organisations’ website updates were used as proxies for the level of activity of each organisation. This choice was consistent with the type of advocacy documents considered for this study: official documents approved by the organisations’ boards. This kind of document is usually uploaded and described on the websites of PHAs. However, PHAs that are mostly active on more informal channels (such as social media), or do not update their websites regularly, might have erroneously been classified as less active in our study.

By choosing to solely analyse officially approved documents, we restricted our review to policies reflecting the official stand of the organisations on different advocacy areas. However, not all advocacy work performed by PHAs is officially approved by the respective boards and general assemblies. As such, the advocacy focus of the PHAs that participated in this study might be wider than represented in our analysis. In addition, language constraints and the fact that the policies were translated using an application may have an impact on the accuracy of the analyses. Future research is needed to assess trends in public health advocacy in these regions to understand how the focus changes over time.

## Conclusion

Overall, our findings reveal that PHAs are active advocates on several public health issues. However, more effort should be devoted to implementing a more international and intersectoral approach, rooted in health as a human right. Better use of resources and evidence to design health interventions, easy accessibility of data for policy and decision-makers, as well as improved funding mechanisms, and citizen awareness are needed to strengthen the advocacy process. In the future, advocacy efforts could involve more academics, journalists, and influencers, who would collaborate with national public health associations and professionals, as a single voice of civil society. The challenge is to raise awareness, build strong networks and alliances, and identify innovative approaches to ensure that policymakers embrace health in all policies and to hold them accountable for the impact of their choices on the health and well-being of the population within and beyond their borders.

## Paper context

Advocacy is one of the core functions of public health. Now more than ever, coordinated, effective, and resilient advocacy is needed at different levels to address health threats such as the COVID-19 pandemic and climate change. In this context, national public health associations play a key role in providing evidence-based recommendations to policymakers. This study aims to understand how advocacy is carried out in different regions and identify strengths and weaknesses in order to assist public health professionals in their work.

## Annex A

**Table A1.** Full list of themes and categories of policy documents.

Themes	Categories (in alphabetical order)
Communicable Diseases	COVID-19 HIV Tuberculosis
Environmental Health	Air pollution/air quality Animal feeding operation Climate change (mental/nutrition/refugees) Ecosystem Energy policy Environmental Land Environmental Noise Environmental Exposure (Asbestos/chemical/Health Risk Assessment) Fossil fuels Mining Occupational Health One health Radioactivity Safe water access Transport & health
Health Equity	Health Inequality Gender Health Geriatric Health Migrant & refugee health Prison health Racism
Human Health Rights	Disability rights Health crisis Health ethics Health Rights (Migrant)
Health System Strengthening	Health Coverage (Universal Health Coverage) Health Investment & Accountability Health surveillance Health system governance Immunisation Long-term Care Primary Health Care Public health infrastructure Solidarity of Public health
Injuries and Violence	Firearm policy/gun violence Harm & violence Worker’s safety
International Health	Antimicrobial resistance Conflict & war Nuclear weapons Trans-Pacific Partnership Trade agreements
Mental Health & Substance Abuse	Gambling Insurance access Mental health wellness Opioid use Sexual violence Substance abuse/Cannabis Suicide prevention
Non-communicable Disease (NCD)	Alcohol control Oral Health Population Ageing Skin cancer Tobacco control/E-cigarette Vision health
Nutrition	Education Excessive Marketing of food/beverages Food labelling Food safety Food security Healthy diet Overweight and obesity
Women & Child Health	Abortion Breast cancer screening Breast feeding child health Oral Care during pregnancy Sexual and reproductive health Women’s health and well-being
